# Shorter Incubation Period among Unvaccinated Delta Variant Coronavirus Disease 2019 Patients in Japan

**DOI:** 10.3390/ijerph19031127

**Published:** 2022-01-20

**Authors:** Tsuyoshi Ogata, Hideo Tanaka, Fujiko Irie, Atsushi Hirayama, Yuki Takahashi

**Affiliations:** 1 Itako Public Health Center of Ibaraki Prefectural Government, Itako 311-2422, Japan; 2 Fujiidera Public Health Center of Osaka Prefectural Government, Fujiidera 583-0024, Japan; TanakaH61@mbox.pref.osaka.lg.jp (H.T.); TakahashiYu@mbox.pref.osaka.lg.jp (Y.T.); 3 Tsuchiura Public Health Center of Ibaraki Prefectural Government, Tsuchiura 300-0812, Japan; f.irie@pref.ibaraki.lg.jp; 4 Department of Public Health and Medical Affairs, Osaka Prefectural Government, Osaka 540-8507, Japan; HirayamaA@mbox.pref.osaka.lg.jp

**Keywords:** COVID-19, delta variant, incubation period, transmission time relative to symptom onset, serial interval, unvaccinated, Japan, SARS-CoV-2

## Abstract

Few studies have assessed incubation periods of the severe acute respiratory syndrome coronavirus 2 Delta variant. This study aimed to elucidate the transmission dynamics, especially the incubation period, for the Delta variant compared with non-Delta strains. We studied unvaccinated coronavirus disease 2019 patients with definite single exposure date from August 2020 to September 2021 in Japan. The incubation periods were calculated and compared by Mann–Whitney U test for Delta (with L452R mutation) and non-Delta cases. We estimated mean and percentiles of incubation period by fitting parametric distribution to data in the Bayesian statistical framework. We enrolled 214 patients (121 Delta and 103 non-Delta cases) with one specific date of exposure to the virus. The mean incubation period was 3.7 days and 4.9 days for Delta and non-Delta cases, respectively (*p*-value = 0.000). When lognormal distributions were fitted, the estimated mean incubation periods were 3.7 (95% credible interval (CI) 3.4–4.0) and 5.0 (95% CI 4.5–5.6) days for Delta and non-Delta cases, respectively. The estimated 97.5th percentile of incubation period was 6.9 (95% CI 5.9–8.0) days and 10.4 (95% CI 8.6–12.7) days for Delta and non–Delta cases, respectively. Unvaccinated Delta variant cases had shorter incubation periods than non–Delta variant cases.

## 1. Introduction

The Delta variant (Phylogenetic Assignment of Named Global Outbreak lineage designation B.1.617.2) is a lineage of severe acute respiratory syndrome coronavirus 2 (SARS-CoV-2). It was first identified in India in late 2020 and was classified as a variant of concern (VOC) on 11 May 2021 [1]. This variant was associated with an estimated increase in transmissibility of 97% [2]. SARS-CoV-2 VOCs bearing the L452R spike protein mutation demonstrate increased transmissibility, infectivity, and avoidance of antibody neutralization [3]. As of 12 October 2021, Delta variant coronavirus disease 2019 (COVID-19) cases had been reported in 191 countries across all six WHO regions. Furthermore, the Delta variant has become the dominant strain in Japan and many other countries [4].

Understanding the transmission dynamics of the virus is important for prediction, interventions, and evaluations of the pandemic. However, knowledge about the transmission dynamics of the Delta variant is insufficient. For example, to the best of our knowledge, only two reviewed articles reported shorter incubation period for the Delta variant [5,6].

Understanding transmission dynamics is important for adequate public health interventions, such as the duration of quarantine, isolation, and contact tracing. The current interventions are based on the findings of transmission dynamics of the SARS-CoV-2 wild-type strain in the early stages of the pandemic. For example, the period of close contact for decisions on the subject of quarantine begins 2 days before symptom onset in a patient in some countries [7,8]. Close contacts are quarantined for 14 days after exposure through contact [7,9,10,11]. Patients with COVID-19 are isolated for 10 days [7,9,11]. These measures are based on the following findings on wild-type strain COVID-19 cases: the period between infection and symptom onset (incubation period) was generally 1–14 days [12,13,14]. Furthermore, the period from the time of symptom onset in the primary patient (infector) to the infector transmitting the virus to a secondary patient (infectee) (transmission time relative to symptom onset) was between two days before and ten days after symptom onset in the infector [15,16]. However, recent studies in Guangdong province, China reported that the Delta variant may have a shorter incubation period than non-Delta variants [5,6]. Thus, current interventions should be discussed based on the transmission dynamics of the Delta variant.

Understanding transmission dynamics is also important for the evaluation of an epidemic. Quantifying the transmission potential before symptom onset will inform epidemic progression predictions [17]. In Japan, cases of Delta variant domestic transmission began to be confirmed in the latter half of May 2021 [18]. The fifth wave of COVID-19, which was primarily caused by the Delta variant, occurred from the latter half of July 2021 to August 2021, with a peak daily number of patients of 25,858 reported on 20 August. The proportion of SARS-CoV-2 virus strains with the L452R mutation was 89% in the week from 16 August to 22 August 2021. However, the daily reported number of patients decreased rapidly after September, as follows: 17,702 on 31 August, 1568 on 30 September, and 147 on 25 October [19,20]. Although the rapid development of vaccines might have contributed to the decline in the number of patients in the fifth COVID-19 wave in Japan, the cause of this rapid decline has not been completely elucidated. Thus, it might be useful to investigate the transmission dynamics of the Delta variant to understand such a decline. While the percentage of pre-symptomatic transmission and transmission time relative to symptom onset might be significant [17,21,22], they could be reduced by public health interventions.

Various factors influence the estimation of the transmission parameters for the Delta variant. In particular, the window of possible exposure in COVID-19 patients is a challenge for assessing estimations [13,23]. Assessing the dynamics of COVID-19 patients with definite single exposure dates would resolve this problem.

With its current predominance, it is necessary to study the transmission dynamics of the Delta variant in order to properly determine public health interventions and understand the mechanism of the pandemic. This study aimed to elucidate the transmission dynamics, especially incubation period, for the Delta variant in comparison with those of non-Delta strains. 

## 2. Materials and Methods

### 2.1. Setting

This study was conducted in the jurisdiction of three public health centers (PHCs) in Japan: Itako PHC, Tsuchiura PHC of the Ibaraki Prefectural Government, and Fujiidera PHC of Osaka Prefectural Government. The jurisdictional areas are located in suburbs in Tokyo and Osaka metropolises and have a total population of approximately 874,000. 

### 2.2. Unvaccinated COVID-19 Cases

This study enrolled individuals who lived in one of the jurisdictions and were confirmed to have SARS-CoV-2 infection, as defined by the relevant PHC. The cases were enrolled for symptom onset from 1 July 2020 through 16 September 2021, and the day of exposure to the virus was specified as a single definite date. We censored enrolment on 30 September 2021.

In Japan, according to the Infectious Diseases Control Law (the law), the PHC must be notified of all COVID-19 cases [7]. SARS-CoV-2 infections were confirmed using polymerase chain reaction (PCR) tests with a cycle threshold value of 40, loop-mediated isothermal amplification tests, antigen quantitative tests, or monoclonal antigen qualitative tests. The PCR test was implemented if the results of any of the other tests were indeterminate. 

The PHC implemented an epidemiological investigation of the patients based on the law. The PHC nurses interviewed the patients and collected data on demographics, symptoms, and history of definite contact with a patient with COVID-19. The onset of symptoms was defined as having any of symptoms including fever (≥37.0 °C), sore throat, headache, and others. The PHC implemented a law-based bidirectional contact tracing of the patients, whether symptomatic or not. Based on the regulations on infectious diseases, the PHC collected PCR test samples from the contacts of index cases. If the first PCR test was negative for a contact, we implemented quarantine of the contact for 14 days from the last exposure. When a contact became symptomatic during quarantine period, we implemented PCR test again.

Among patients, we extracted COVID-19 cases for whom we could estimate a definite single date of exposure. In most cases, the COVID-19 infectee had contact with the COVID-19 infector on a single date and was not in any other transmission setting. We also included COVID-19 patients in a cluster with no less than three COVID-19 patients and were not in other transmission settings. In these cases, the infector could not be identified, and the exposure date was that of the event when the cluster occurred. We excluded patients with complete vaccination, which we defined as symptom onset that had passed not less than 14 days after the second vaccination. Number of patients with completely vaccination was expected to be very small. The cumulative number of confirmed COVID-19 cases per population in the jurisdiction of the three PHCs was 0.61% at the end of June 2021 and 1.33% at the end of September 2021; therefore, we assumed that contacts were susceptible to SARS-CoV-2 infection.

### 2.3. Participant COVID-19 Cases with Delta Variant and Non-Delta Strains

Among unvaccinated COVID-19 patients with a definite single date of exposure, we determined the following patients with the Delta variant strain: cases reported from 8 July to September 2021 and in which the L452R variant was detected in the patients or their contacts. In Japan, screening for the L452R mutation was implemented for approximately 40–50% of samples from July to August 2021. The L452R mutation was also found in other variant strains of interest, such as the B.1.617.1 (Kappa) variant. However, almost all cases with the L452R mutation in Japan were confirmed to be the Delta variant by RNA sequencing. For example, the domestic number of VOCs confirmed by genome sequencing was 42,721 for B.1.617.2 (Delta), 47,856 for B.1.1.7 (Alpha), and 8 for B.1.617.1 (Kappa) as of 27 September [19].

Among unvaccinated COVID-19 patients with a definite single date of exposure, we defined non-Delta strain cases as either those reported from November 2020 to 6 June or those reported after 7 June and with L452R mutation negative results for not less than two people among the patients and their contacts. No L452R variant had been confirmed in COVID-19 cases reported until 7 June 2021 in these areas [24,25]. Because the result of L452R mutation was sometimes false negative, we thought that we could not classify a patient as non-Delta in case only one L452R variant was negative in the patient or among their contacts.

If a case was reported after 7 June, and, in addition, L452R mutation screening was not performed for the patient, or was all negative, or only one L452R variant was negative in the patient or among their contacts as described above, the patient was excluded from the study.

### 2.4. Statistical Analysis

The periods between viral exposure and symptom onset (incubation period), symptom onset date of the infector and that of infectee (serial interval), and the symptom onset date for the infector and the exposure date for the infectee (transmission time relative to symptom onset) were calculated in the Delta variant and non-Delta strain patient groups.

We implemented Mann–Whitney U test for comparison of parameters between Delta strain and non-Delta strain, and we defined statistical significance as *p* < 0.05. 

We fitted the parametric distribution of Gaussian, Gamma, Lognormal and Weibull to the data, and calculated parameters and 95% credible interval (95% CI) of the distribution with the smallest value of Akaike’s information criterion (AIC) on Delta patient group in the Bayesian statistical framework. We adopted the identical distribution for fitting to data of non-Delta patients. Because data on serial interval and transmission time relative to symptom onset included non-positive value, we shifted data by adding 3 days to each serial interval and adding 5 days to each transmission time relative to symptom onset so that we could fit distribution [26].

We estimated and compared mean and percentiles of incubation period by fitting Lognormal distribution among SARS-CoV-2 Delta and non-Delta patients. In Delta variant cases, mono-variable estimates of means of incubation periods were calculated by fitting distribution, using factors such as sex, age, infector and/or patient eating at exposure, and exposure setting. The transmission settings were classified as restaurants, schools, houses, and other contact settings. The transmission in house included both that in household members and that in friends.

Statistical analyses were performed using R (version 4.4-1; R Foundation for Statistical Computing, Vienna, Austria).

### 2.5. Ethical Approval

The study protocol was approved on 26 October 2019, by the Osaka University Hospital Observational Research Ethics Review Committee (protocol number: T20114). Active epidemiological investigation data analyses were performed in accordance with the Infectious Diseases Control Law, and the study was exempted from the requirement for informed consent under “the ethical guidelines for life science and medical research on human subjects” in Japan.

## 3. Results

In total, 232 COVID-19 patients had one definite date of exposure to the virus. Only 8 patients out of them were completely vaccinated, and they were excluded from the participants. We enrolled 224 unvaccinated COVID-19 patients with one definite date of exposure to the virus, as follows: 121 patients with the Delta strain and 103 with non-Delta strains. The characteristics of the participants are listed in Table 1.

Table 2 shows number of participants and results of Mann–Whitney U test for comparison of parameters of transmission dynamics between SARS-CoV-2 Delta strain and non-Delta strain. In 120 symptomatic patients with the L452R variant from 72 infectors, the mean incubation period was 3.7 days, and in 100 symptomatic patients with non-Delta strains, the mean incubation period was 4.9 days; the difference was significant (*p*-value = 0.000). The mean serial interval was 2.8 days in 88 patients with the Delta variant and 3.3 days in 66 non-Delta variant cases; the difference was not significant. 

The transmission time relative to symptom onset was distributed between −4 and 7 days in 94 patient pairs with the Delta variant, and it was negative (i.e., transmitted at least one day before the date of onset) in 63 (67%) patient pairs. The mean transmission time relative to symptom onset was −0.94 days. In 66 non-Delta patient pairs, the transmission time relative to symptom onset was distributed between −4 days and 4 days, and it was negative in 53 (80%) patient pairs. The mean transmission time relative to symptom onset was −1.39 days; the difference was significant by Mann–Whitney U test (*p* = 0.012).

We implemented sensitive analyses by Mann–Whitney U test for participants excluding those with incomplete vaccination. Mean incubation period of participants with zero vaccination was 3.7 days among 104 Delta variant patients and 4.9 days among 99 non-Delta variant patients; the difference was significant (*p*-value = 0.000). Transmission time relative to onset was −0.90 days among 80 Delta variant patients with zero vaccination and −1.36 days among 62 non-Delta variant patients with zero vaccination; the difference was significant (*p*-value = 0.020).

**Figure 1:**
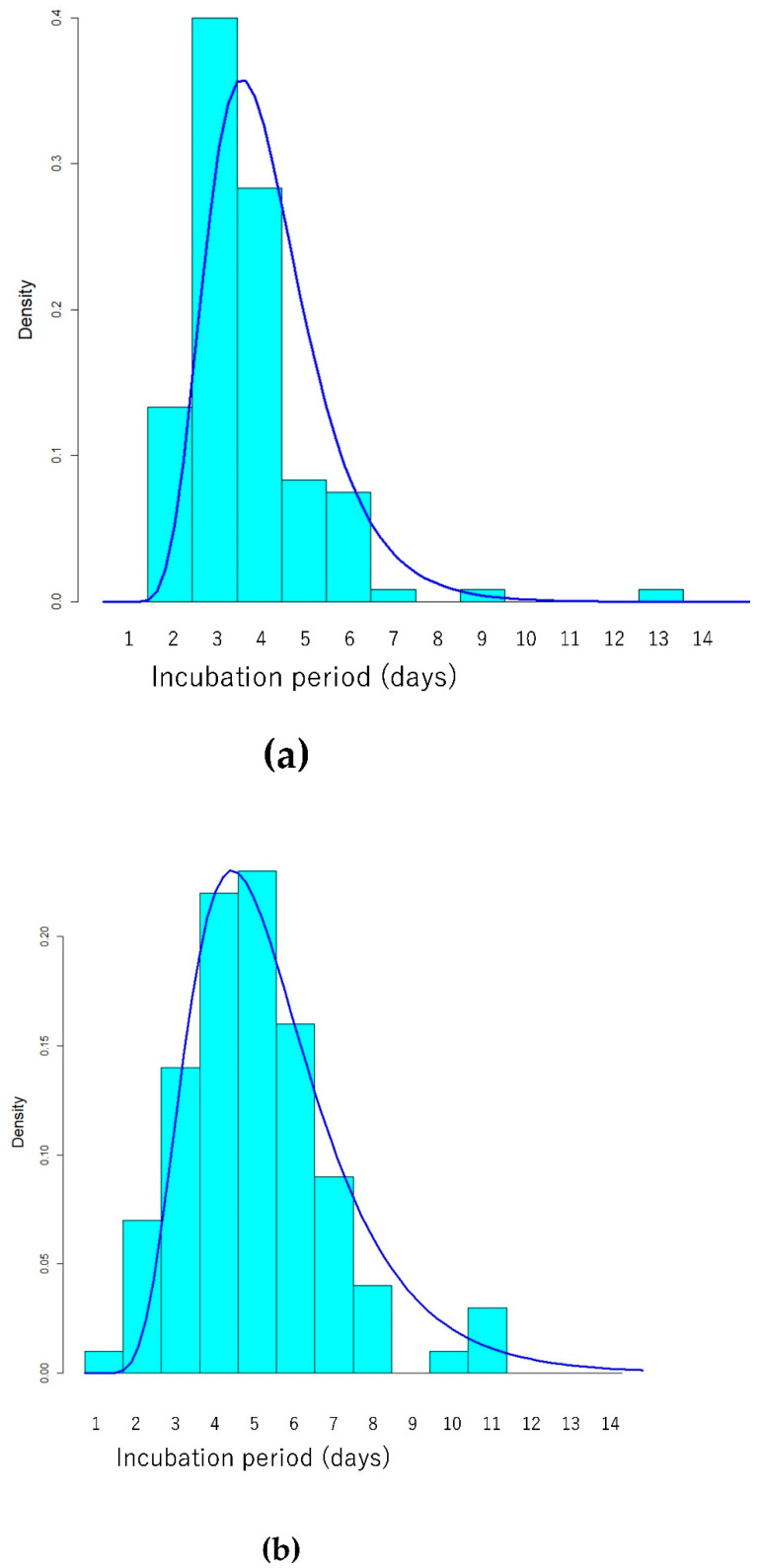

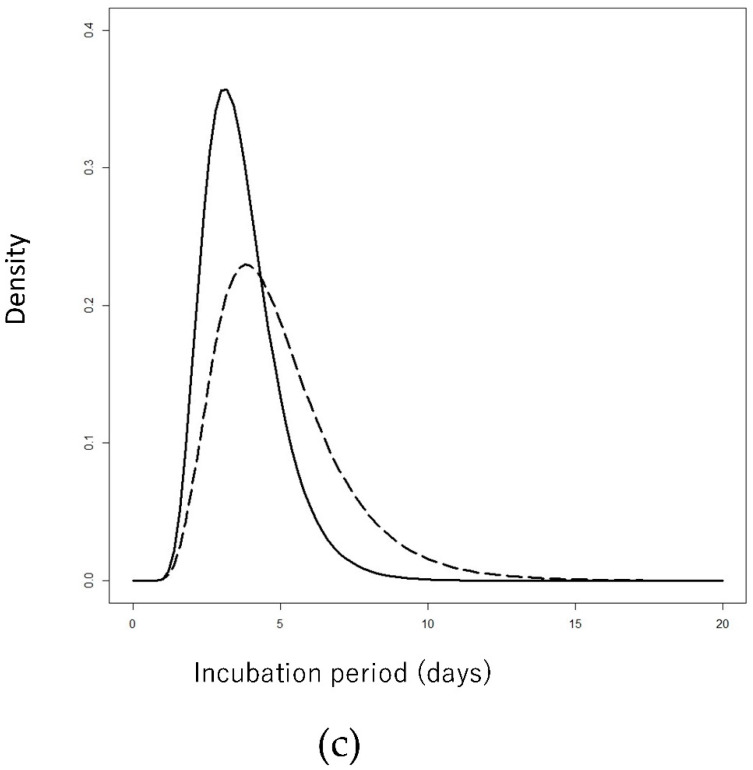
Histogram and probability density of estimated Lognormal distribution of incubation period (days) for (**a**) Delta and (**b**) non-Delta cases in Japan. (**c**) Comparison of probability density of estimated Lognormal distribution of incubation periods between Delta (solid line) and non-Delta cases (dashed line).

**Figure 2:**
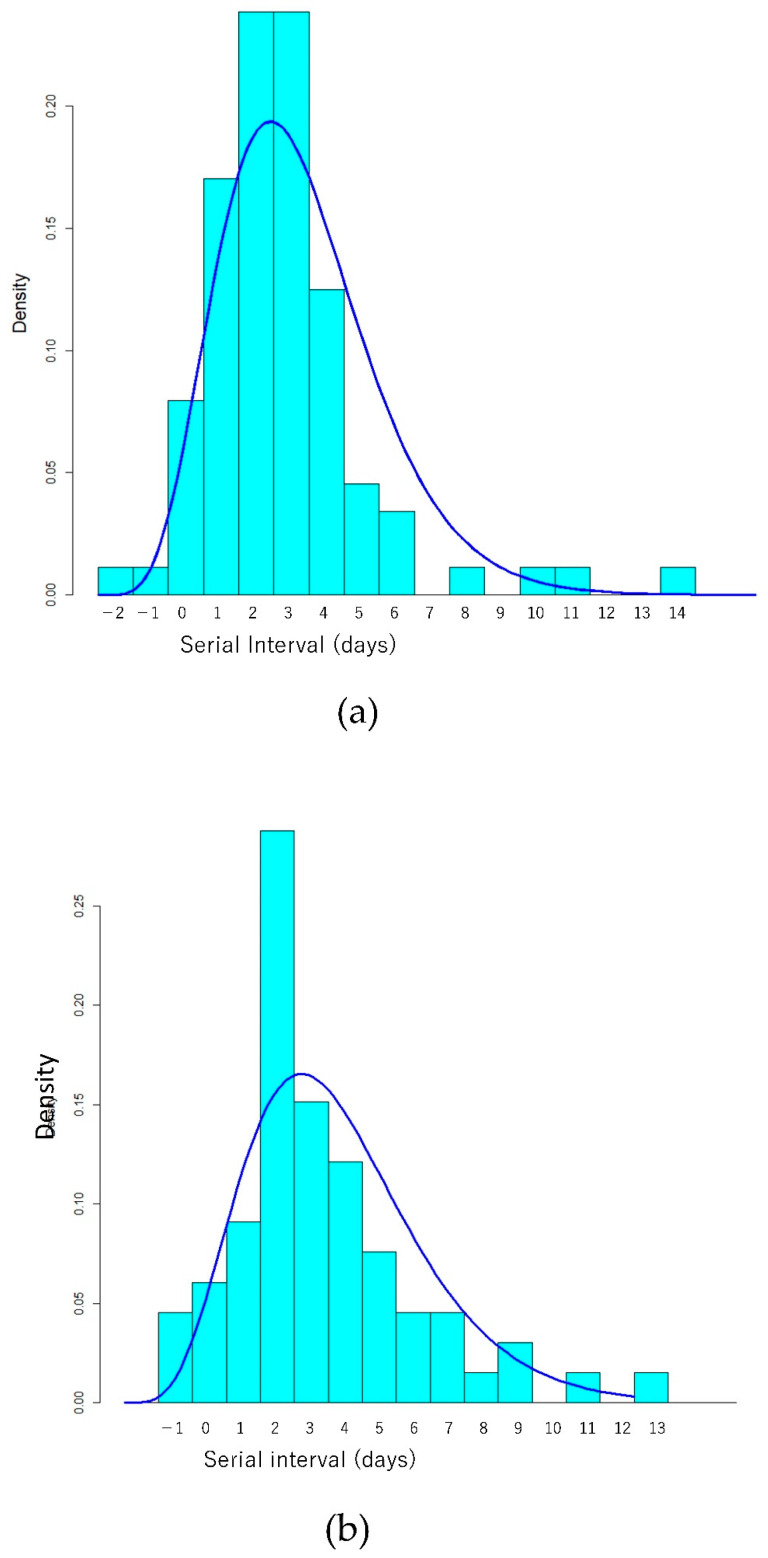

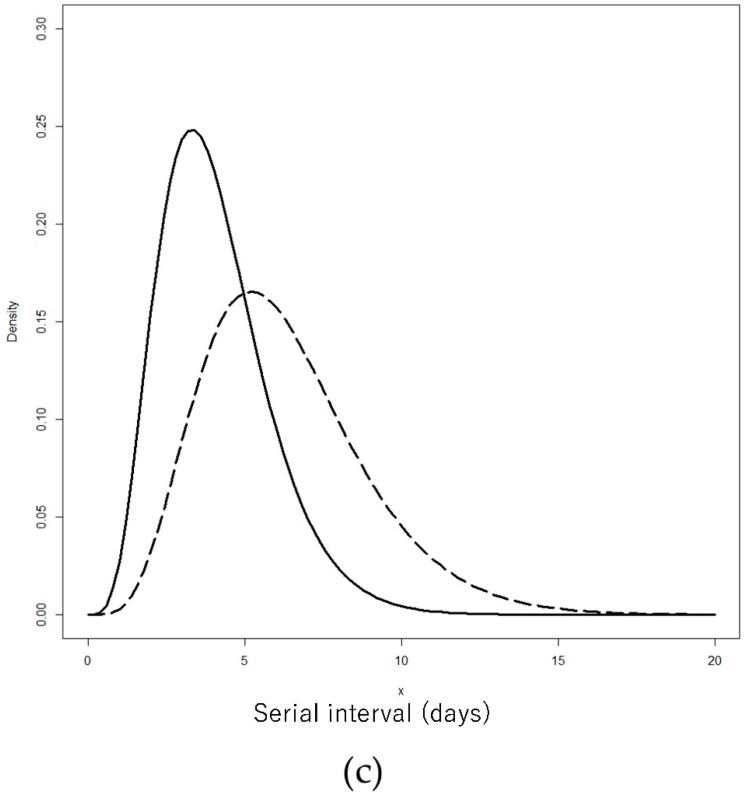
Histogram and probability density of estimated Gamma distribution of serial interval for (**a**) Delta and (**b**) non-Delta cases. (**c**) Comparison of probability density of estimated Gamma distribution of shifted (adding 3 days to data) serial interval between Delta (solid line) and non-Delta cases (dashed line).

**Figure 3:**
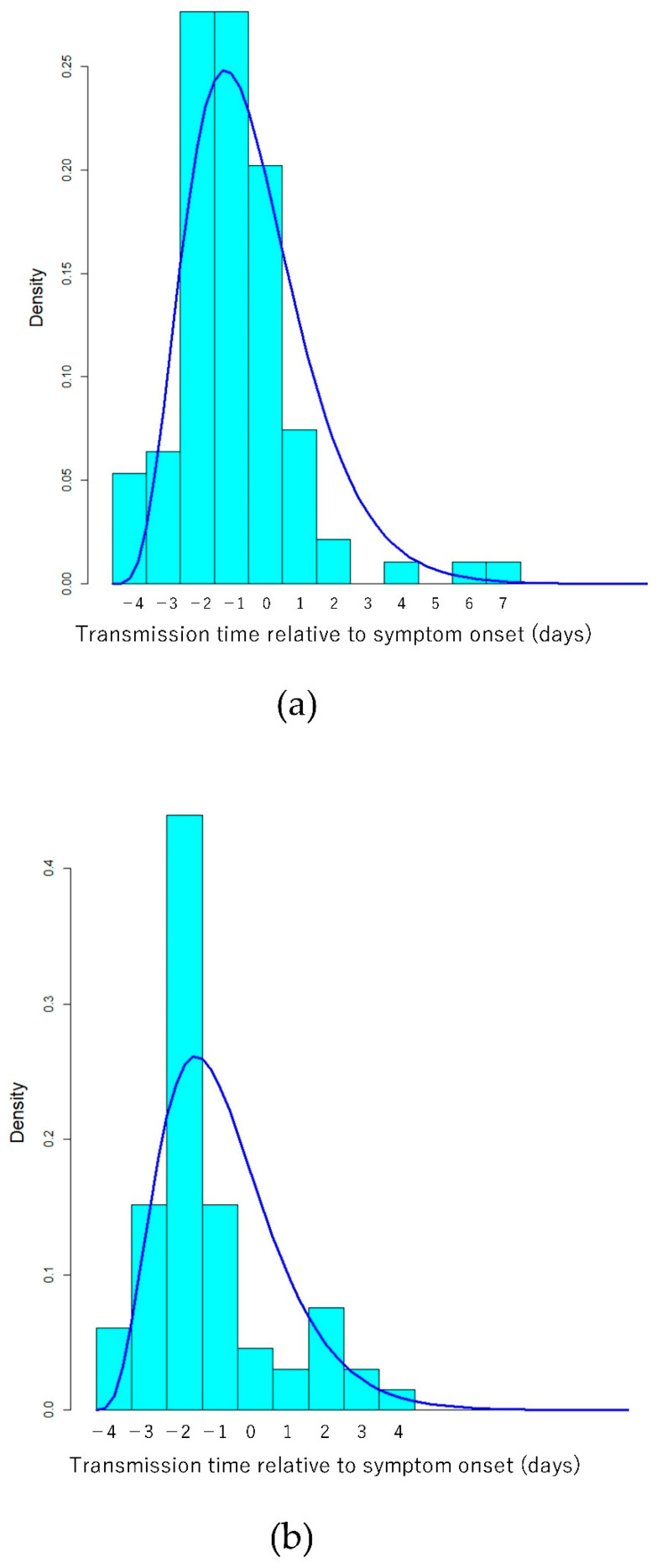

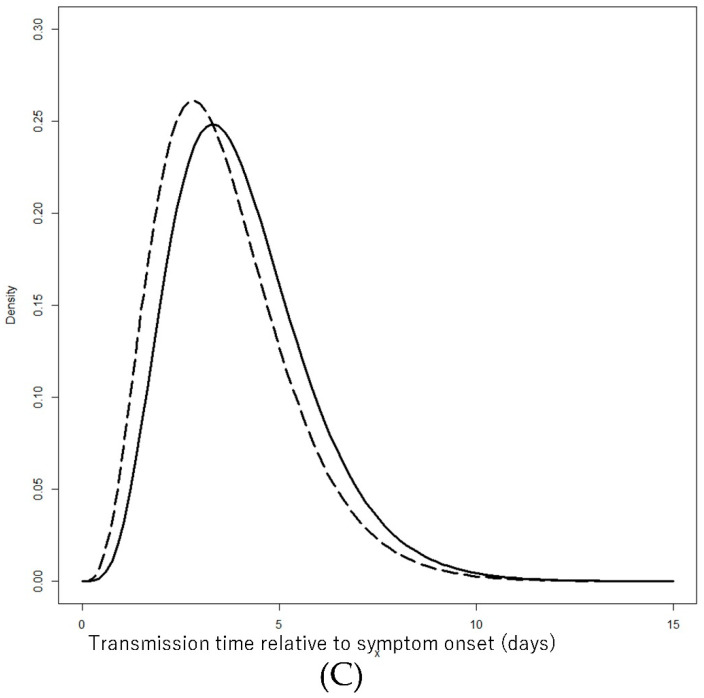
Histogram and probability density of estimated Gamma distribution of transmission time relative to symptom onset for (**a**) Delta and (**b**) non-Delta cases. (**c**) Comparison of probability density of estimated Gamma distribution of shifted (adding 5 days to data) transmission time relative to symptom onset between Delta (solid line) and non-Delta cases (dashed line).

Table 3, Figure 1, Figure 2 and Figure 3 show the estimated parameters for SARS-CoV-2 transmission dynamics.

When the Lognormal distribution, whose Akaike’s information criterion (AIC) was smaller than that of Gaussian, Gamma, and Weibull distributions, was fitted to the data, the estimated mean of incubation period among Delta variant patients was 3.7 (95% CI 3.4–4.0) days, the estimated median was 3.5 (95% CI 3.3–3.7) days, and the 97.5th percentile of incubation period was estimated to be 6.9 (95% CI 5.9–8.0) days (Table 4 and Figure 1a).

The estimated mean incubation period was 5.0 (95% CI 4.5–5.6) days, and the estimated median incubation period was 4.6 (95% CI 4.2–5.0) days among non–Delta variant patients when the Lognormal distribution was fitted (Table 4 and Figure 1b). Therefore, the estimated mean incubation period was significantly shorter for Delta variant cases (3.7 days [95% CI 3.4–4.0]) than for non–Delta strain cases (5.0 days [95% CI 4.5–5.6]) (Figure 1c). The estimated incubation period of the 97.5th percentile was 10.4 (95% CI 8.6–12.7) days.

The estimated mono-variable mean incubation period by fitting Lognormal distribution among Delta variant patients did not differ significantly with sex, age, eating at contact, and contact setting (Table 5).

We implemented sensitive analyses for participants excluding those with incomplete vaccination. When the Lognormal distribution, whose Akaike’s information criterion (AIC) was smaller than that of Gaussian, Gamma, and Weibull distributions, was fitted to the data, the estimated mean of incubation period among 104 Delta variant patients with zero vaccination was 3.7 (95% CI 3.4–4.1) days, the estimated median was 3.5 (95% CI 3.3–3.7) days, and the 97.5th percentile of incubation period was estimated to be 7.1 (95% CI 6.1–8.5) days. Among 99 non–Delta variant patients with zero vaccination, the estimated mean incubation period was 4.9 (95% CI 4.5–5.6) days, the estimated median incubation period was 4.5 (95% CI 4.2–5.0) days, and the estimated 97.5th percentile of incubation period was 10.3 (95% CI 8.7–12.9) days when the Lognormal distribution was fitted. Therefore, the estimated mean incubation period was significantly shorter for Delta variant cases than for non–Delta strain cases.

## 4. Discussion

In the present study, the Delta variant strain had a shorter incubation period compared with non-Delta strains.

The incubation period for the Delta variant was 3.7 days, which was significantly shorter than that for non-Delta strains. The incubation period did not significantly differ with the factors analyzed. To the best of our knowledge, only three peer-reviewed studies in Guangdong province, China, reported the incubation period (4.0, 4.4, and 6.0 days) in Delta variant cases [5,6,27]. These incubation periods were shorter [5,6] or longer [27] than those of non-Delta cases. The present study supports the former results.

The upper limit of the estimated incubation period of the 97.5th percentile was approximately 8 days in the present study. In Japan and other countries, close contacts have been quarantined for 14 days [7,9,10,11]. However, Delta variant cases have a short incubation period, and currently vaccination promotes the coexistence of humans with SARS-CoV-2 rather than the “zero-COVID” strategy. Therefore, it may be appropriate for unvaccinated close contacts of COVID-19 patients to shorten the quarantine period to, for example, approximately 8 days. 

The mean serial interval for the Delta variant was 2.8 days and was not significantly shorter than that for non-Delta variants in the present study. In the previous literature, it was 2.3 days (mean) in China, 3 days (median) in Singapore, and 3.3 days (mean) in Korea [5,28,29]. Although these figures are apparently shorter than those in previous reports for the wild-type strain [30,31], they could have been reduced by various interventions, such as self-isolation, quarantine of close contacts, and lockdown [26,32]. 

The transmission time relative to symptom onset was distributed between −4 and 7 days in this study. Contact tracing and quarantine have been implemented for close contacts with exposure to the virus 2 days before symptom onset in the infector patient or later in several countries [7,8]. However, it may be necessary to expand subject close contacts for tracing and quarantine to those with exposure to the virus three and four days before symptom onset in the infector patient. 

In the Delta variant group of the present study, viral transmission was not observed after the eighth day after symptom onset in the infector patient. The viral load after symptom onset may be higher in Delta variant cases than in non-Delta cases [5,33]. However, the present study did not support extending the currently recommended 10-day patient isolation period [33].

In the present study, most of the transmission occurred during the infector’s pre-symptomatic period. These findings are consistent with those of previous studies [21,22]. The proportion of pre-symptomatic transmission may be an important indicator because pre-symptomatic and asymptomatic transmission may play an important role [34]. While transmission after symptom onset in infector patients can be reduced by interventions, such as voluntary quarantine after symptom onset, it is generally difficult to prevent transmission from asymptomatic or pre-symptomatic infectors. Therefore, the proportion of pre-symptomatic transmission could be reduced by interventions and time varying.

In this study, patients with the Delta variant strain had a shorter transmission time before symptom onset (1.4 days) than those with non-Delta strains (2.0 days) by Mann–Whitney U test. In previous studies on the wild-type strain, the transmission time relative to symptom onset varied among countries [17]. Nonetheless, the transmission time relative to symptom onset could be reduced by voluntary quarantine after symptom onset. On the contrary, it is difficult to influence the transmission time before symptom onset using such interventions. Compared with non-Delta strains, the Delta-variant may have a shorter duration of transmission to contacts before symptom onset. 

The fifth wave of COVID-19 in Japan, which occurred in the latter half of July 2021, was mainly caused by the Delta variant. Compared with non-Delta variant cases, Delta variant cases may have higher viral shedding [5,33], resulting in a higher basic reproduction number. If intervention is inadequate, the transmission and number of patients with the Delta variant may increase sharply in the area. However, the number of patients in Japan decreased continuously after September. Although the rapid development and roll-out of vaccines has contributed to the decline in the number of patients in the fifth wave in Japan, the cause of this rapid decline has not been completely elucidated. Possible reasons for the rapid decline include the relatively recent timing of vaccination, mainly in July and August, continuous observance of public health interventions, such as mask wearing [20], and climate change [35]. In Japan, the rapid increase in the number of COVID-19 cases in the early stage of the fifth wave might have promoted patient behavior aimed at avoiding transmission, such as self-quarantine and undergoing COVID-19 testing after symptom onset. Therefore, in the later stage of the fifth wave, the shorter pre-symptomatic transmission period, as described above, indicating higher proportion of behaviorally avoidable transmission, in addition to other factors, might be a factor in the sharp decline in the number of cases.

This study had several limitations. First, the relatively small number of participant cases compared to the total number of patients in the area might have caused selection bias [14,36]. However, we adopted similar procedures and estimation methods for both Delta and non-Delta cases, which might have partially offset the bias. Second, the syndromic case definition may have influenced the outcome period, compared to other studies [14,37]. Third, censoring of follow-up observations and truncation might have influenced the selection of participants and outcome period [23]. Fourth, the Delta variant was mainly confirmed by the L452R mutation. However, genome sequencing showed coincidence between the Delta variant and L452R mutation in Japan [19].

In the future, it is necessary to further study transmission dynamics and live viral shedding, including in vaccinated patients, household attack rate [27,38], and genomic analysis of the Delta variant. It is also necessary to continue the surveillance of epidemiological data and other VOCs including the Omicron variant.

## 5. Conclusions

Patients with the Delta variant strain had a shorter incubation period and a shorter period of transmission of the virus to contacts before symptom onset, compared with those with non-Delta strains.

## Figures and Tables

**Table 1:** Characteristics of participants.

Variables	*N*
Total	224
Sex	
Male	156
Female	68
Age	
≤29	119
≥30	105
Delta mutation	
Delta	121
Non-Delta	103

**Table 2:** Results of Mann–Whitney U test for comparison of parameters of transmission dynamics between SARS-CoV-2 Delta strain and non-Delta strain.

Parameters	Delta		Non-Delta		Mann–Whitney U Test
	N	Mean	N	Mean	*p*-Value
		(Days)		(Days)	
Incubation period	120	3.7	100	4.9	0.000
Serial interval	88	2.8	66	3.3	0.227
Transmission time relative to onset	94	−0.94	66	−1.39	0.012

SARS-CoV-2 = severe acute respiratory syndrome coronavirus.

**Table 3:** Estimated parameters for SARS-CoV-2 transmission dynamics.

Parameters	Fitted Distribution	Delta		Non-Delta	
		Meanlog/Shape	Sdlog/Rate	Meanlog/Shape	Sdlog/Rate
		Days (95% CI)	Days (95% CI)	Days (95% CI)	Days (95% CI)
Incubation period	Lognormal (Meanlog/Sdlog)	1.25 (1.19–1.31)	0.34 (0.30–0.39)	1.52 (1.43–1.60)	0.42(0.37–0.48)
Serial interval (added 3 days)	Gamma (Shape/Rate)	7.1 (5.1–9.5)	1.24 (0.88–1.67)	6.1 (4.2–8.2)	0.97 (2.6–4.0)
Transmission time relative to onset (added 5 days)	Gamma (Shape/Rate)	5.5 (4.0–7.3)	1.37 (0.98–1.81)	4.7 (3.3–6.3)	1.32(0.90–1.80)

CI = credible interval; SARS-CoV-2 = severe acute respiratory syndrome coronavirus 2.

**Table 4:** Estimated mean and percentiles of incubation period by fitting Lognormal distribution among SARS-CoV-2 Delta and non-Delta patients.

Indicators	Delta	Non-Delta
	Days (95% CI)	Days (95% CI)
Mean	3.7 (3.4–4.0)	5.0 (4.5–5.6)
Median	3.5 (3.3–3.7)	4.6 (4.2–5.0)
2.5th percentile	1.8 (1.5–2.1)	2.0 (1.6–2.4)
97.5th percentile	6.9 (5.9–8.0)	10.4 (8.6–12.7)

**Table 5:** Estimated incubation period by fitting Lognormal distribution among SARS-CoV-2 Delta variant by variables.

Variables		Mean	Mono-Variable Mean Estimate (95% CI)
	*N*	(Days)	(Days)
Total	120	3.7	3.7 (3.4–4.0)
Sex			
Male	82	3.6	3.7 (3.4–4.0)
Female	38	3.8	3.9 (3.3–4.6)
Age			
≤29	77	3.6	3.6 (3.3–4.0)
≥30	43	3.9	3.9 (3.4–4.5)
Eating of infector and/or patient at exposure			
Yes	58	3.9	3.8 (3.4–4.4)
No or unknown	62	3.6	3.6 (3.3–3.9)
Setting			
Restaurant	33	3.9	3.9 (3.2–4.8)
School	29	3.8	3.8 (3.2–4.6)
House	22	3.8	3.8 (3.3–4.6)
Others	36	3.4	3.4 (3.1–3.9)

## Data Availability

The data presented in this study are available upon reasonable request from the corresponding author. The data are not publicly available because of the protection of personal information.
